# You must not fool yourself: Feynman, neurodiversity, and honest AI in digital mental health

**DOI:** 10.3389/fdgth.2026.1819411

**Published:** 2026-07-14

**Authors:** David Ruttenberg

**Affiliations:** UCL Institute of Education, University College London, London, United Kingdom

**Keywords:** AI ethics, artificial intelligence, cognitive diversity, digital mental health, Feynman, honesty, neurodiversity, predictive processing

## Abstract

Digital mental health is undergoing rapid transformation through the deployment of artificial intelligence (AI) systems including conversational agents, risk-prediction tools, and clinical decision-support platforms. Despite growing enthusiasm, the field has yet to converge on an operational standard for what constitutes honest, trustworthy AI in psychiatric and psychological contexts—one that explicitly accounts for the limits, blind spots, and populations for whom deployed systems have not been validated. This Perspective draws on Richard Feynman's foundational principle of scientific integrity—articulated in his 1,974 Caltech commencement address, “Cargo Cult Science”—and Carl Sagan's dimensional metaphors to develop a practical, disclosure-oriented ethical framework for AI in digital mental health. The neurodiversity paradigm provides a critical stress test: growing empirical evidence indicates that AI tools trained on narrow behavioural and linguistic norms carry substantial risk of misrepresenting neurodivergent users, with the potential to silently re-encode stigma, misclassification, and inequity into clinical systems. Drawing on predictive processing theory and recent empirical evidence of AI bias against neurodivergent populations, this work proposes five criteria for the Feynman Honesty Standard: scope clarity, population-limit disclosure, uncertainty communication, role integrity, and participatory co-development. The analysis concludes with a forward-looking research agenda for clinicians, developers, and policymakers, arguing that the field will be judged not only by algorithmic performance but by its candour about what these systems know, miss, and cannot do.

## 1 Introduction

Artificial intelligence is reshaping digital mental health at a pace that has outrun our ethical frameworks. Conversational agents are being deployed as therapeutic companions, predictive algorithms risk-stratify patients without their knowledge, and generative AI produces clinical summaries and care plans at scale (1, 2). The promise is genuine: AI could democratise access to mental health support and surface patterns invisible to unaided judgment (3). Yet the central ethical question is not whether AI works, but whether we are honest about what it knows, what it misses, and who it harms (4, 5).

Existing ethical frameworks for AI in mental health have made genuine progress. Convergent scholarship has established core principles of autonomy, beneficence, non-maleficence, and justice as the normative foundation for responsible deployment (5–7). Regulatory and professional guidance has begun to operationalise transparency, accountability, and uncertainty communication as requirements rather than aspirations (4, 8). What these frameworks have not yet delivered is one oriented specifically around candour—one that foregrounds not just what AI systems should disclose, but the institutional and epistemic honesty demanded of the researchers, developers, and clinicians who build and deploy them. An honesty standard asks whether the field is telling the whole truth about what its systems know and cannot know, and about who absorbs the cost when that truth is withheld (9).

This Perspective proposes a disclosure-oriented synthesis: a clinical sharpening of existing transparency commitments, organised around Feynman's principle of radical self-honesty and tested against the specific vulnerabilities of neurodivergent users (9–11). The argument that such a standard already exists in embryonic form in Feynman's concept of cargo cult science—which maps with uncomfortable precision onto current AI deployment in mental health—is not a claim of novelty but a provocation toward honest self-examination (9, 12). It should be noted at the outset that the empirical evidence through which this framework is stress-tested addresses primarily autism and ADHD; the extent to which findings generalise to other neurodevelopmental conditions is addressed explicitly in Section 3.

This argument proceeds in four steps. First, Feynman's first principle is translated into actionable criteria for AI integrity in digital mental health. Second, the neurodiversity paradigm is employed as a stress test, using empirical evidence of AI bias against neurodivergent populations to examine where the field risks fooling itself (10, 11). Third, Carl Sagan's dimensional metaphors and predictive processing theory are used to reframe AI tools as constrained projections rather than objective truths (13, 14). Finally, this work proposes a five-criterion Feynman Honesty Standard and a forward research agenda.

## 2 Feynman's first principle and cargo-cult risks in digital mental health

In his 1,974 commencement address at the California Institute of Technology—later published as “Cargo Cult Science” in Caltech's Engineering and Science magazine—Richard Feynman articulated the foundational principle of scientific integrity: “The first principle is that you must not fool yourself—and you are the easiest person to fool” (9). He described cargo cult science as work carrying the superficial appearance of scientific rigour—controlled studies, performance metrics, peer-reviewed publications—without the deeper commitment to honest self-examination that distinguishes genuine inquiry from institutional performance. The cargo cult scientist reports results selectively, builds on methodological assumptions without testing them, and mistakes statistical outputs for understanding (9, 12).

The resonance with current AI development in digital mental health is uncomfortable and exact. Conversational AI platforms have been marketed as “clinically validated” on the basis of narrow pilot studies conducted in non-representative populations (1, 2). Risk-stratification algorithms have been deployed in crisis services with performance metrics measured on historical data that encodes existing clinical biases (5, 15). Generative AI produces outputs that are fluent and contextually plausible, creating the illusion of clinical understanding without a genuine model of the person being served (3, 16). In each case, the field risks repeating Feynman's cargo cult: assembling the instruments of evidence-based medicine without its animating commitment to honest self-scrutiny (9, 12).

In mental health, the stakes of self-deception are amplified by patient vulnerability and the power asymmetries embedded in clinical relationships (6). This vulnerability is particularly acute for neurodivergent populations, who experience substantially higher rates of co-occurring conditions: up to 68% of autistic individuals present with at least one additional psychiatric diagnosis, with depression and anxiety affecting approximately 25% and 27% respectively, and suicide risk elevated three to six times compared to neurotypical populations (17–19).

These individuals carry histories of diagnostic delay, misclassification, and institutional harm that predate the current wave of AI deployment—burdens documented independently of any AI system (20, 21). AI systems deployed without neurodivergent validation do not introduce risk into a previously neutral landscape; they amplify and encode inequities that are already structural (10, 11, 22). If the field does not apply Feynman's standard—acknowledging what AI cannot do, for whom it has not been designed, and where failures will land hardest—consequences will not be abstract ethical violations but real clinical harm to real people (4, 16).

## 3 Neurodiversity as a stress test for honest AI

The neurodiversity paradigm holds that neurological variation—including autism, attention-deficit/hyperactivity disorder (ADHD), dyslexia, and related conditions—represents natural and valuable human diversity rather than deviation from a pathological norm (23, 24). If AI systems are trained predominantly on neurotypical behavioural norms, they will not simply fail to serve neurodivergent users—they will actively misrepresent them, encoding pathology where there is difference (10, 11).

The empirical basis for this concern is growing at three distinct levels. At the level of general-purpose LLM encoders, peer-reviewed studies document significant implicit bias against neurodivergence-related terms across multiple model architectures, with bias persisting even for autistic traits—such as honesty and directness—where positive representation might reasonably be expected (10, 23). At the level of NLP classification models applied in mental health and psychiatry, systematic bias has been identified at every stage of model development—in training data, architecture, and clinical interpretation—with none of the 52 studies reviewed in one landmark analysis having addressed all sources of potential bias simultaneously (22, 24). At the level of deployed conversational agents, emerging evidence documents harm pathways including emotional dependency, amplification of internalised stigma, and near-complete absence of regulatory accountability or safety standards (25). These three levels are analytically distinct: LLM encoder bias does not confirm equivalent harm in deployed clinical tools, and neither directly establishes the magnitude of harm in real service settings. What the evidence establishes is that the concern is not theoretical—and that systematic, population-specific evaluation has yet to be mounted.

Neurodivergent users therefore constitute a rigorous stress test for the Feynman Honesty Standard. A system not evaluated against neurodivergent populations, that has not disclosed its performance gaps, and that has not involved neurodivergent communities in its design cannot claim to meet Feynman's first principle (9–11). Neurodevelopmental conditions are among the most prevalent reasons people seek mental health support, and AI tools that silently re-encode deficit-based framings of autism or ADHD risk serious harm to populations already bearing disproportionate mental health burdens (17, 18, 26, 27).

The empirical evidence reviewed here addresses primarily autism and ADHD, where the research literature is most developed. The extent to which conclusions generalise to other neurodevelopmental conditions—including dyslexia, dyscalculia, developmental language disorder, and Tourette syndrome—remains an open question requiring dedicated investigation. Parallel concerns regarding AI bias in dyslexia assessment are emerging and noted where relevant, but the evidentiary base is thinner and conclusions should not be extrapolated freely (22, 24).

A further gap deserves explicit acknowledgment: the developmental. The literature drawn on here is predominantly adult-based—mental health burden data (17–19), service-user experience data (20), and conversational agent evidence (25) all address adult populations. Adolescents are among the most frequently reported informal users of conversational AI for emotional support, often outside clinical settings; emotional dependency pathways (25) may be heightened during adolescent development. Participatory co-development with neurodivergent young people raises questions of assent, parental involvement, and age-appropriate design not addressed by the adult-focused co-design literature currently cited. A dedicated developmental extension of this framework is acknowledged as a priority for future investigation.

## 4 Dimensional metaphors, predictive processing, and the limits of AI perception

Carl Sagan employed the metaphor of dimensional constraint to illuminate a fundamental epistemological limit: a two-dimensional being inhabiting Flatland cannot perceive a three-dimensional object passing through its plane—only a cross-section, a distorted shadow (13, 28). The most dangerous assumption any observer can make is that this constrained vantage point captures the whole. Applied to AI in digital mental health, this metaphor reframes a critical risk: when AI tools are treated as higher-dimensional observers of the human mind, clinicians and patients may mistake a constrained projection for privileged truth.

Predictive processing theory in cognitive neuroscience provides a rigorously grounded parallel. The brain is modelled as a hierarchical prediction machine: perception is not passive reception but active construction in which prior models shape what we detect, amplify, and suppress (14, 29). AI systems similarly function as constrained projection architectures, mapping data onto prior distributions shaped by their training history (14, 29). The parallel has limits as important as the similarity: human clinicians are embodied, morally accountable agents whose priors are subject to iterative revision through direct encounter, collegial challenge, and the lived consequences of their judgements (14). AI models apply training priors without the corrective apparatus that clinical judgment, at its best, can bring to bear (10, 24).

For digital mental health, this has a direct practical implication: AI outputs should be presented not as assessments but as projections—constrained cross-sections shaped by the specific dimensional limitations of training and design. Honest AI communicates not just its outputs but the shape of its blind spots (5, 9, 13). This reframing does not diminish the value of AI tools; it locates them correctly within a clinical epistemology that Sagan and Feynman would both recognise: one that is humble about what any single instrument can see.

## 5 Toward a Feynman honesty standard for AI in digital mental health

Building on established principles of transparency, accountability, and care (4–6, 8) and on the foregoing analysis, this work proposes five operational criteria for the Feynman Honesty Standard in digital mental health. These criteria are intended not as a replacement for existing frameworks but as a practical, implementable extension—a translation of Feynman's first principle into the specific context of AI-mediated psychiatric and psychological care.

The five criteria are as follows:

Scope clarity: Systems must provide explicit, user-facing statements of what they are not designed or validated to do—excluding clinical diagnosis, risk determination without human oversight, or therapeutic intervention from stated capabilities unless specifically validated in the target population (4, 5, 8).

Population-limit disclosure: Systems must document and disclose the demographic, neurological, linguistic, and developmental characteristics of training and validation populations—including age and developmental stage, gender, race, and neurodevelopmental status—with explicit statements of expected performance degradation for under-represented groups including neurodivergent, gender-diverse, minority-language, non-Western, and paediatric or adolescent users (10, 11, 30).

Uncertainty communication: Outputs relevant to risk, prognosis, or clinical decision-making must be accompanied by user-intelligible uncertainty indicators—plain-language expressions of what the system does not know and the range of error associated with its outputs, not merely technical confidence scores in documentation (14, 29).

Role integrity: System design, interface language, and documentation must maintain clear distinctions between support, triage, and treatment. Conversational AI must not be positioned—through naming, persona design, or marketing—as a clinician, therapist, or diagnostic authority. Anthropomorphisation inviting clinical trust without clinical accountability violates Feynman's first principle (1, 3, 16, 25). In practice, role integrity can be assessed through: audits of product materials against a documented function statement; alignment with Software as a Medical Device (SaMD) classification frameworks including the FDA AI/ML SaMD Action Plan (2021) (31) and the NICE Evidence Standards Framework for Digital Health Technologies (2022) (32); and pre-deployment review of capability claims against independently verified technical documentation, flagging anthropomorphic framing as a specific audit category.

Participatory co-development: Neurodivergent individuals and other affected communities must be structurally involved—not merely consulted—in defining acceptable uncertainty, identifying harm scenarios, and setting performance standards for AI tools used in their care (10, 11, 27, 33). This criterion can be operationalised using the GRIPP2 checklist (34) for standardised reporting of patient and public involvement, with additional mechanisms including pre-registration of co-design protocols, disaggregated demographic reporting by neurodevelopmental condition and age group, and documented community advisory board outputs with traceable influence on design decisions.

Together, these criteria operationalise Feynman's central demand: that engineers, clinicians, and policymakers commit to telling the whole truth about what they know and do not know (9). They are measurable through documentation review, interface audit, and structured community consultation, making them tractable for regulatory guidance and journal reporting standards.

Honesty failures in digital mental health AI are structurally produced. Commercial development has proceeded at a pace outrunning population-validated evidence, driven by investor timelines and the competitive logic of first-mover advantage in a rapidly expanding market (5, 22). Market incentives reward fluency, novelty, and scaling—none of which require transparency about populations for whom a system has not been validated. The accountability gap around AI companion products marketed for mental health and social support contexts remains largely unenforced (25). Costs are not borne symmetrically: developers capture the benefit of adoption while neurodivergent individuals—over-routed into AI-mediated systems and under-represented in the training data those systems deploy—absorb the risk of misclassification and harm (10, 22). The Feynman Honesty Standard is a structural corrective: a demand that the burden of disclosure be redistributed from the most vulnerable users to the institutions profiting from deployment, using directionally consistent but methodologically heterogeneous evidence as its mandate.

The regulatory landscape is not uniformly undeveloped: the EU AI Act [Regulation (EU) 2024/1689], which entered into force in August 2024, subjects AI systems deployed in healthcare contexts to high-risk classification requirements, imposing obligations on transparency, human oversight, and technical documentation (35); and the WHO's 2024 guidance on ethics and governance of large AI models in health establishes foundational requirements for explainability and equity (36). What these frameworks do not yet provide are population-specific disclosure requirements for neurodivergent users, operational definitions of role integrity in conversational AI, or developmental stage-specific provisions. The Feynman Honesty Standard is proposed as a practitioner-facing complement to, not substitute for, regulatory compliance.

## 6 Implications and a forward research agenda

For clinicians, the Feynman Honesty Standard demands a recalibrated interpretive stance toward AI outputs in mental health practice. A clinician who accepts an AI risk-stratification score without interrogating population-limit disclosures or uncertainty bounds is, in Feynman's terms, engaged in cargo cult medicine (9, 15). Clinical training should incorporate AI literacy as a core competency—not merely how to use AI tools, but how to read their limitations documentation, challenge their outputs, and communicate uncertainty to patients, including those with neurodivergent communication styles (8, 37).

For developers, the criteria require an epistemological reorientation: from optimising performance metrics on benchmark datasets to designing for honest failure. A system that clearly communicates what it cannot do, and for whom it has not been designed, trained, and tested, is more clinically trustworthy than one that maximises accuracy on a non-representative test set while obscuring its limitations (5, 10, 16).

For policymakers and journal editors, the Standard translates directly into reporting requirements. Journals publishing AI-in-mental-health research should require explicit disclosure of training population characteristics, neurodiversity representation, uncertainty quantification, and participatory design evidence as conditions of publication (4, 6, 8). Regulatory frameworks should mandate population-limit disclosure and treat Feynman Honesty Standard compliance as prerequisite for clinical deployment, consistent with high-risk AI classification requirements under existing frameworks (35, 36). The three research priorities below provide the empirical foundation this policy agenda requires.

## 7 Conclusion

Feynman's first principle was not a counsel of paralysis but a prerequisite for genuine progress. A science that does not fool itself is the only science capable of actually learning. Applied to AI in digital mental health, this principle yields a question the field can no longer responsibly defer: are we telling the whole truth—to developers, clinicians, regulators, patients, and neurodivergent communities—about what these systems know, what they do not know, and who bears the cost of their errors? (9).

A growing and directionally consistent body of evidence regarding AI bias risk against neurodivergent populations makes clear that we are not yet answering that question honestly (10, 11). The dimensional metaphors of Sagan and the predictive processing frameworks of cognitive neuroscience remind us that AI outputs represent constrained projections of human complexity, and that the most dangerous errors in clinical AI are those we have mistaken for objectivity (13, 14, 29). The five criteria proposed here offer a tractable path from these philosophical commitments to practical change in how AI tools in digital mental health are built, documented, regulated, and used (4–6, 8).

Three research priorities emerge as most urgent: the development of neurodiversity-inclusive validation datasets for mental health AI—including paediatric and adolescent populations, and conditions beyond autism and ADHD where validation data are most sparse; the operationalisation of the Feynman Honesty Standard into standardised regulatory and reporting checklists; and longitudinal study of whether honest-AI practices produce measurably better equity and trust outcomes for neurodivergent mental health service users. Digital mental health will be judged not only by the performance of its algorithms but by whether the field had the integrity to tell the truth about them (9, 11, 16).

## Data Availability

The original contributions presented in the study are included in the article/Supplementary Material, further inquiries can be directed to the corresponding author.

## References

[1] Luxton DD. Recommendations for the ethical use and design of artificial intelligent care providers. Artif Intell Med. (2014) 62(1):1–10. 10.1016/j.artmed.2014.06.004 25059820

[2] Gaffney H Mansell W Tai S. Conversational agents in the treatment of mental health problems: mixed-method systematic review. JMIR Ment Health. (2019) 6(10):e14166. 10.2196/14166 31628789 PMC6914342

[3] Stade EC Stirman SW Ungar LH Boland CL Schwartz HA Yaden DB. Large language models could change the future of behavioral health: a proposal for responsible development and evaluation. NPJ Ment Health Res. (2024) 3:12. 10.1038/s44184-024-00056-z 38609507 PMC10987499

[4] Torous J Myrick KJ Rauseo-Ricupero N Firth J. Digital mental health and COVID-19: using technology today to accelerate the curve on access and quality tomorrow. JMIR Ment Health. (2020) 7(3):e18848. 10.2196/18848 32213476 PMC7101061

[5] Martinez-Martin N Dasgupta I Carter A Chandler C Kellmeyer P. Ethics of digital mental health: an umbrella review. J Med Internet Res. (2020) 22(3):e17314. 10.2196/17314

[6] Vayena E Blasimme A Cohen IG. Machine learning in medicine: addressing ethical challenges. PLoS Med. (2018) 15(11):e1002689. 10.1371/journal.pmed.1002689 30399149 PMC6219763

[7] Beauchamp TL Childress JF. Principles of Biomedical Ethics. 8th ed Oxford: Oxford University Press (2019).

[8] American Psychological Association. Ethical Guidance for the use of Artificial Intelligence in the Professional Practice of Health Service Psychology. Washington DC: APA (2025). (Accessed 2026 February 27). Available online at: https://www.apa.org/topics/artificial-intelligence-machine-learning/ethical-guidance-ai-professional-practice

[9] Feynman RP. Cargo cult science. Eng Sci. (1974) 37(7):10–3. Available online at: https://calteches.library.caltech.edu/51/2/CargoCult.htm (Accessed July 03, 2026).

[10] Brandsen S Chandrasekhar T Franz L Grapel J Dawson G Carlson D. Prevalence of bias against neurodivergence-related terms in artificial intelligence language models. Autism Res. (2024) 17(2):234–48. 10.1002/aur.3094 38284311 PMC12233132

[11] Rizvi N Strickland H Gitelman D Morales Flores A Cooper T Kallepalli A. AUTALIC: a dataset for anti-autistic ableist language in context. In: Proceedings of the 63rd Annual Meeting of the Association for Computational Linguistics (Volume 1: Long Papers); 2025. Vienna, Austria: Association for Computational Linguistics (2025). p. 20999–1015. 10.18653/v1/2025.acl-long.1022

[12] Simmons JP Nelson LD Simonsohn U. False-positive psychology: undisclosed flexibility in data collection and analysis allows presenting anything as significant. Psychol Sci. (2011) 22(11):1359–66. 10.1177/0956797611417632 22006061

[13] Sagan C. Cosmos. New York: Random House (1980).

[14] Clark A. Whatever next? Predictive brains, situated agents, and the future of cognitive science. Behav Brain Sci. (2013) 36(3):181–204. 10.1017/S0140525X12000477 23663408

[15] Obermeyer Z Powers B Vogeli C Mullainathan S. Dissecting racial bias in an algorithm used to manage the health of populations. Science. (2019) 366(6464):447–53. 10.1126/science.aax2342 31649194

[16] Skjuve M Følstad A Fostervold KI Brandtzaeg PB. My chatbot companion – a study of human-chatbot relationships. Int J Hum Comput Stud. (2021) 149:102601. 10.1016/j.ijhcs.2021.102601

[17] Hollocks MJ Lerh JW Magiati I Meiser-Stedman R Brugha TS. Anxiety and depression in adults with autism spectrum disorder: a systematic review and meta-analysis. Psychol Med. (2019) 49(4):559–72. 10.1017/S0033291718002283 30178724

[18] Hirvikoski T Mittendorfer-Rutz E Boman M Larsson H Lichtenstein P Bölte S. Premature mortality in autism spectrum disorder. Br J Psychiatry. (2016) 208(3):232–8. 10.1192/bjp.bp.114.160192 26541693

[19] Culpin I Mars B Pearson RM Hammerton G Heron J Carpenter P. Autistic traits and suicidal thoughts, plans, and self-harm in late adolescence: population-based cohort study. J Am Acad Child Adolesc Psychiatry. (2018) 57(5):313–20. 10.1016/j.jaac.2018.01.023 29706160 PMC5942156

[20] Adams D Malone S Lawson W Breasley J Girdler S Carroll A. Autistic people’s experiences with mental health services: intersectionality matters. Neurodiversity. (2025). 10.1177/27546330251369785

[21] Srivarathan A Bradford A Shearkhani S Heimlich L Jefferson S Miller KE. Bridging diagnostic safety and mental health: a systematic review highlighting inequities in autism spectrum disorder diagnosis. BMJ Qual Saf. (2025). Advance online publication. 10.1136/bmjqs-2025-018723 PMC12707049 40854799

[22] Timmons AC Duong JB Fiallo NS Lee T Vo HPQ Ahle MW. A call to action on assessing and mitigating bias in artificial intelligence applications for mental health. Perspect Psychol Sci. (2023) 18(5):1062–96. 10.1177/17456916221134490 36490369 PMC10250563

[23] Venkit PN Srinath M Wilson S. A study of implicit bias in pretrained language models against people with disabilities. In: Proceedings of the 29th International Conference on Computational Linguistics (COLING 2022); 2022 Oct 12–17. Gyeongju, Republic of Korea: International Committee on Computational Linguistics (2022). p. 1324–34. Available online at: https://aclanthology.org/2022.coling-1.113

[24] Straw I Callison-Burch C. Artificial intelligence in mental health and the biases of language based models. PLoS One. (2020) 15(12):e0240376. 10.1371/journal.pone.0240376 33332380 PMC7745984

[25] Papadopoulos C. The use of AI chatbots for autistic people: a double-edged sword of digital support and companionship. Neurodiversity. (2025). 10.1177/27546330251370657

[26] Singer J. Neurodiversity: The Birth of an Idea. Self-published (2016).

[27] Chapman R. Neurodiversity and the social ecology of mental functions. Synthese. (2021) 198(Suppl 19):4349–66. 10.1007/s11229-020-02748-9 33577400

[28] Abbott EA. Flatland: A Romance of Many Dimensions. London: Seeley (1884).

[29] Kiverstein J Miller M Rietveld E. The feeling of grip: novelty, error dynamics, and the predictive brain. Synthese. (2017) 196(7):2847–69. 10.1007/s11229-017-1583-9

[30] Fusaroli R Lambrechts A Bang D Bowler DM Gaigg SB. Is voice a marker for autism spectrum disorder? A systematic review and meta-analysis. Autism Res. (2017) 10(3):384–407. 10.1002/aur.1678 27501063

[31] U.S. Food and Drug Administration. Artificial Intelligence/Machine Learning (AI/ML)-Based Software as a Medical Device (SaMD) Action Plan. Silver Spring, MD: FDA (2021). Available online at: https://www.fda.gov/media/145022/download (Accessed July 03, 2026).

[32] National Institute for Health and Care Excellence (NICE). Evidence Standards Framework for Digital Health Technologies. London: NICE (2022). Available online at: https://www.nice.org.uk/about/what-we-do/our-programmes/evidence-standards-framework-for-digital-health-technologies (Accessed July 03, 2026).

[33] Ruttenberg DP. Towards technologically enhanced mitigation of autistic adults’ sensory sensitivity experiences and attentional, and mental wellbeing disturbances. Phd thesis, University College London, London (2025). Available online at: https://discovery.ucl.ac.uk/id/eprint/10210135/ (Accessed July 03, 2026).

[34] Staniszewska S Brett J Mockford C Barber R Burnard P Chadwick GL. GRIPP2 Reporting checklists: tools to improve reporting of patient and public involvement in research. Br Med J. (2017) 358:j3453. 10.1136/bmj.j3453 28768629 PMC5539518

[35] European Parliament and Council of the European Union. Regulation (EU) 2024/1689 of the European parliament and of the council of 13 June 2024 laying down harmonised rules on artificial intelligence (artificial intelligence act). Off J Eur Union. (2024) L 2024/1689. Available online at: https://eur-lex.europa.eu/legal-content/EN/TXT/?uri=OJ:L_2024_1689 (Accessed July 03, 2026).

[36] World Health Organization. Ethics and Governance of Artificial Intelligence for Health: Guidance on Large Multimodal Models. Geneva: WHO (2024). Available online at: https://www.who.int/publications/i/item/9789240084759 (Accessed July 03, 2026).

[37] Topol EJ. High-performance medicine: the convergence of human and artificial intelligence. Nat Med. (2019) 25(1):44–56. 10.1038/s41591-018-0300-7 30617339

